# Potential impacts of a future Nordic bioeconomy on surface water quality

**DOI:** 10.1007/s13280-020-01355-3

**Published:** 2020-09-12

**Authors:** Hannu Marttila, Ahti Lepistö, Anne Tolvanen, Marianne Bechmann, Katarina Kyllmar, Artti Juutinen, Hannah Wenng, Eva Skarbøvik, Martyn Futter, Pirkko Kortelainen, Katri Rankinen, Seppo Hellsten, Bjørn Kløve, Brian Kronvang, Øyvind Kaste, Anne Lyche Solheim, Joy Bhattacharjee, Jelena Rakovic, Heleen de Wit

**Affiliations:** 1 Water, Energy and Environmental Engineering Research Unit, University of Oulu, P.O. Box 4300, 90014 Oulu, Finland; 2 Finnish Environment Institute, Latokartanonkaari 11, 00790 Helsinki, Finland; 3 Natural Resources Institute Finland, University of Oulu, P.O. Box 413, 90014 Oulu, Finland; 4 Norwegian Institute of Bioeconomy Research (NIBIO), Fredrik A. Dahls vei 20, 1430 Ås, Norway; 5 Department of Soil and Environment, Swedish University of Agricultural Sciences, Box 7014, 75007 Uppsala, Sweden; 6 Norwegian University of Life Science, Ås, Norway; 7 Swedish University of Agricultural Sciences, Box 7050, 75007 Uppsala, Sweden; 8 Finnish Environment Institute, University of Oulu, P.O. Box 413, 90014 Oulu, Finland; 9 Department of Bioscience, Aarhus University, Vejlsøvej 25, 8600 Silkeborg, Denmark; 10 Norwegian Institute for Water Research, Gaustadalléen 21, 0349 Oslo, Norway; 11 University of Agder, Pb 422, 4604 Kristiansand, Norway; 12 Finnish Environment Institute SYKE, Freshwater Centre, Latokartanonkaari 11, 00790 Helsinki, Finland; 13 Norwegian Institute of Bioeconomy Research (NIBIO), P.O. Box 115, 1431 Ås, Norway

**Keywords:** Bioeconomy, Land use, Surface water, Water quality

## Abstract

Nordic water bodies face multiple stressors due to human activities, generating diffuse loading and climate change. The ‘green shift’ towards a bio-based economy poses new demands and increased pressure on the environment. Bioeconomy-related pressures consist primarily of more intensive land management to maximise production of biomass. These activities can add considerable nutrient and sediment loads to receiving waters, posing a threat to ecosystem services and good ecological status of surface waters. The potential threats of climate change and the ‘green shift’ highlight the need for improved understanding of catchment-scale water and element fluxes. Here, we assess possible bioeconomy-induced pressures on Nordic catchments and associated impacts on water quality. We suggest measures to protect water quality under the ‘green shift’ and propose ‘road maps’ towards sustainable catchment management. We also identify knowledge gaps and highlight the importance of long-term monitoring data and good models to evaluate changes in water quality, improve understanding of bioeconomy-related impacts, support mitigation measures and maintain ecosystem services.

## Increased demand for biomass is a challenge to sustainable management of Nordic surface waters

Clean surface and coastal waters are hallmarks of the Nordic countries. These waters are valuable natural resources and essential for Nordic societies, economies and human wellbeing as they provide multiple ecosystem services. Nordic catchments have varying climates and geohydrology which influence agricultural and forest productivity. These catchments currently face multiple stressors, e.g. increasing demand for resources, increased production levels, global warming and changes in the hydrological cycle. The EU bioeconomy strategy states that more wood and crop-based biomass is needed to move towards a low-carbon and resource-efficient society in which fossil resources are replaced by renewables to mitigate climate change (European Commission 2012). This societal transformation towards a more circular bio-based economy, known as the ‘green shift’ from use of fossil fuels towards renewable resources, is expected to increase the demand for biomass production in the Nordic countries and elsewhere in Europe. The current understanding of the consequences, especially for water systems, is limited (Golembiewski et al. 2015). Land use practices will most likely change, the volume of biomass extracted will increase and this will likely influence water quality (Rosegrant et al. 2013) and the local hydrology. Land use changes predicted for the Nordic countries include intensified forestry (Eyvindson et al. 2018) and crop production (Jordan et al. 2007), perhaps also on marginal soils such as peatlands, which will increase the pressure on water systems.

Trade-offs between ecosystem services are inevitable, since the increased need for biomass has adverse effects on the biodiversity and provision of ecosystem services, and these effects are eventually reflected in the structure and function of freshwater ecosystems (Jordan et al. 2007). In the Nordic countries, threats to freshwater ecosystems and coastal seas include eutrophication (Bechmann and Stålnacke 2019), brownification (e.g. de Wit et al. 2016) and biodiversity loss (Riemann et al. 2016). The impacts of bioeconomic development on environmental goals are addressed by international strategies that aim at halting biodiversity loss (European Union 2011), limiting the global temperature rise (IPCC Paris Agreement), and obtaining good ecological and chemical status of all surface water bodies within the European Union (EU) and the EFTA countries (Water Framework Directive (WFD) 2000). These strategies, however, may not be compatible with increased biomass production and removal. Compromises are therefore needed when taking multiple, often controversial, objectives into account in land use planning and decision making (Juutinen et al. 2019). In natural resources planning and policy making, benefits are usually evaluated separately from each other, which overlooks their potential trade-offs. This generally leads to overexploitation of natural resources.

The WFD and many national regulations implementing the WFD aim for good ecological, and chemical status for all surface water bodies by the end of 2027. There are many concerns that river basin management and restoration actions are too slow to reach the WFD aims and that diffuse nutrient loadings, in particular, should be diminished significantly (e.g. Hering et al. 2010; Räike et al. 2019). There are increasing demands for solutions that balance land management practices and water body ecological goals, while simultaneously considering multiple uses of land, water and ecosystem services (Giri and Qiu 2016; Johansen et al. 2018).

Although we do not yet fully appreciate the possible changes in land use following a transition to the bioeconomy, some conclusions can be drawn based on projected global futures (Rakovic et al. 2020). One likely scenario is intensified land use pressures in all Nordic countries. At the same time, it must be acknowledged that Nordic countries have a great variation in land use patterns both in terms of agriculture and forestry (Table 1, Hagen et al. 2013). Furthermore, the crops produced, dominant forest tree species and soils all differ across the Nordic region. For example, Finland has about one-third of their forests on peatlands, whereas Norwegian and Swedish forests mainly are on moraine deposits and mineral soils. Nevertheless, there are also many similarities between the Fennoscandian countries, having a generally cool climate and large areas with boreal forests with rather low level of human activities, as well as thousands of rivers and lakes, which is much more than the rest of Europe. These natural characteristics create a large potential for bioeconomic development, but also for negative impacts on the many relatively pristine rivers and lakes. Denmark is very different from the other Nordic countries and more comparable to Central-European countries (e.g. Northern Germany, the Netherlands) having a milder climate, very few areas with low human activities due to a large proportion being used for agriculture, and has mainly very small lakes and many small streams.

**Table 1:** Proportions of land cover in Nordic countries

	Denmark	Finland	Norway	Sweden
Agriculture (%)	63.4	7.5	3.5	7.5
Forest (%)	12.9	72.9	37.4	68.7
Other (%)^a^	23.7	19.6	59.1	23.8

^a^Peatland, freshwater, mountain areas above the treeline, open uncultivated land, urban areas, open areas (in the north minor vegetation, in the south bedrock), etc

In the following, we highlight the importance of considering the water quality effects of increasing biomass extraction from Nordic catchments, focusing on Norway, Finland, Sweden and Denmark. The aim of this paper is to highlight the water quality aspects of the ‘green shift’ in a Nordic perspective, assess the state of the understanding, and identify knowledge gaps with focus on suitability of existing monitoring and modelling tools for Nordic landscapes.

## Anthropogenic activities that impact water quality in Nordic catchments

Nordic surface waters have many specific challenges that need to be considered and have been identified as posing a risk to sustaining good ecological status, in particular:

- Intensive agricultural practices causing leaching of phosphorus and nitrogen, and erosion processes especially on silty, clayey and other vulnerable soil types. Coastal areas with acid sulphate soil deposits and risk for downstream acidification. Agricultural cultivation of peatlands leading to leaching of nutrients and carbon.
- Intensification of forestry both in peatlands and on mineral soils. Peatland forestry with drainage and soil preparation, and intensification of forest harvesting (especially clear-cutting and whole-tree harvesting), leading to leaching of organic carbon and nitrogen, and loss of base cations.
- Increased pressure from increased nutrient and carbon loads on coastal and inland waters and drinking water reservoirs. Surface water and groundwater of high quality and several ecosystem services.
- Regional variations of management practices, catchment vulnerability to disturbances and expected climate change (multiple stressors), challenging predictions of land use on water quality.

In the following sections, we treat these challenges in a Nordic perspective.

### The agricultural sector in Nordic countries and its impact on water quality

For centuries, Nordic countries have created climate-adapted and optimised cultivation systems for food security. Losses of suspended sediments, nitrogen and phosphorus from these systems to surface waters vary widely between catchments (Pengerud et al. 2015; Tattari et al. 2017). Environmental factors like geology and climate significantly influence the water quality and ecological status, e.g. sloping clay soils contribute high levels of suspended solids and phosphorus, while coarser soils with a high content of organic matter cause high levels of nitrogen loss (Bechmann et al. 2008). Drainage of coastal clay deposit formed in the Litorina sea stage contains sulphate that leaches as sulphuric acid resulting in low pH and release of metals (Fältmarsch et al. 2008). Production intensity is also a major factor influencing agriculture pollution and loads to receiving waters (Bechmann et al. 2008). From an agricultural perspective, societal demands for more biomass would imply intensification of production and clearing of new land for agriculture (Stehfest et al. 2010). For decades, cheap fertilisers prices have led to high use of phosphorus (P) and accumulation of P in soils. Also utilisation of marginal land areas for e.g. paludiculture may increase leaching. This adds new pressures on watercourses and potentially increases pollutant loading.

Changes in agricultural management over the past decades have led to increased intensity of production in some farming regions in the Nordic countries, while in other regions farming is becoming more extensive and agricultural land is being abandoned (Bechmann et al. 2008). These trends will probably accelerate in the future. Arable land with fertile soils in climate regions that are favourable for agricultural production will be more intensively managed. Higher productivity may increase the risk for elevated leaching rates if, e.g. crops are destroyed due to more extreme weather. Agricultural land close to riparian areas may also be used for more water retention measures such as wetlands, ponds, vegetated buffer zones, irrigation reservoirs and floodplains. This will further increase the need for efficient use of remaining agricultural land for food production.

Soil erosion and losses of particulate phosphorus are the main threats to water quality in regions with a high proportion of clay soil (Ulén et al. 2010), but also nitrogen losses from clay soils can be considerable (e.g. Tattari et al. 2017). Nitrogen losses is a severe problem for farmers and coastal waters in areas with coarser soils especially if agricultural production is intensive with high fertilisation rates. In Finland, agriculture is also conducted on peatland/organic soils, where the high organic matter content with low C/N ratios causes carbon and nitrogen leaching to water courses (Räike et al. 2019). Drained, organic soils offer a good growth substrate, but release high loads of nutrients to water courses. In recent decades, water protection measures and mitigation to reduce environmental impacts of food production have gained more attention, in part because of the EU WFD. The effects of mitigation measures under the EU WFD are difficult to assess but appear to vary widely (Stålnacke et al. 2014; Pengerud et al. 2015). The WFD ‘fitness check’ (EC 2019) concludes that substantial progress in water bodies’ overall status is lacking, but the reason is difficult to assess because of many changes in monitoring and classification methodologies preventing a confident comparison of the two classification cycles (EEA 2018). These methodological changes include adding more eutrophication-sensitive biological quality elements like benthic algae in rivers and taking hydromorphological quality elements into account in assessment. In addition, the effect of climate on nutrient runoff is a confounding factor for assessment of mitigation measures on water quality, which illustrates the need for process-based understanding of catchment-scale processes.

Although challenges in evaluation of the extent and the efficiency of the mitigation measures implemented, clear evidence of changes in waters is shown for some areas in the Nordic countries. The strict Danish policy actions has resulted in a significant reduction in nitrogen loss from Danish agriculture (Dalgaard et al. 2014). In Sweden, nitrogen leaching has decreased in regions where mitigation measures such as catch crop, spreading of manure in spring instead of autumn and spring ploughing has been implemented to a large extent (Fölster et al. 2012). Also the agricultural advisory campaign focus on nutrients has most likely contributed to better nitrogen efficiency in these regions. In Norway the effect of mitigation measures is not so visible because of changes in climate and management at the same time (Bechmann et al. 2019; Lyche Solheim et al. 2020). The opposite also occurs, e.g. one recent study from Finnish river basins shows that the effects of water protection measures are not visible in total N and total P loads due to changes in precipitation and temperatures (Räike et al. 2019).

Sustainable crop production must include measures to minimise soil disturbance and reduce negative effects on water quality (Ulén et al. 2010). Soil compaction by farm machinery has been shown to cause decreased infiltration and increased runoff risk in agricultural land areas (Seehusen et al. 2019). Soil tillage is the main agricultural practice causing soil disturbance and increased risk of erosion and nutrient losses (Ulén et al. 2010). Tillage in autumn, as opposed to spring, leaves soil uncovered during a long period of the year when runoff occurs. However, fields without autumn tillage are a bigger source of dissolved phosphorus (Puustinen et al. 2007) as vegetation cover transport phosphate from deeper layers to the top surface. New cropping systems will be required to further decrease the nutrient leaching. Vegetation that is permanent or at least covers the soil during most of the year is here a key factor. Catch crops succeeding the main crop is effective against nitrogen leaching but the effect on leaching of phosphorus is more unclear (Aronsson et al. 2016). Careful selection of crop production systems and avoidance of bare soil layers in fields are key to achieve a successful sustainable bioeconomy in agricultural regions. In drained areas, controlled drainage systems offer ways to the sustain water table and minimise leaching, e.g. from cultivated peatlands. In general, good cropping conditions maintain growth and plant nutrient uptake limiting leaching potential. In Nordic conditions, drainage might be important to prevent water logging.

There is a risk that the political push towards a bioeconomy may cause riparian zones and other marginal lands to be more intensively utilised for fuel, fodder and food. At the same time, natural riparian zones can be highly useful in keeping soil particles, nutrients and other pollutants from entering the water bodies and thus should be protected and maintained in areas of intensive agriculture (Stutter et al. 2019). Restoring and maintaining natural vegetation in riparian zones will provide buffer zones where nutrients can infiltrate in the soil and be used by the terrestrial vegetation, and thereby reduce losses to the water bodies (Turunen et al. 2019). Riparian zones also have many important ecological functions (Tolkkinen et al. 2020).

### The forestry sector in the Nordic countries and its impacts on water quality

Finland, Norway and Sweden have a strong tradition of commercial forestry and use of wood-based products. However, limited empirical data are available on the impacts of a forest-based bioeconomy on waters, particularly in the longer term in the whole Nordic region. More intensified forestry might include afforestation on new areas, densification of existing forests, fertilisation prior to harvest and a move from stem-only harvest towards whole-tree-harvest to produce biofuels that can replace fossil fuels (see, e.g. Futter et al. 2019). Further, continuous cover forestry, e.g. in Finland is becoming more popular in order to avoid harmful effects of clear cuttings. The sustainability of forest management is currently debated. Evidence of increased exports of carbon, nutrients and suspended solids to water courses following forest harvest is quite well established (e.g. Kreutzweiser et al. 2008). Many studies report temporary nutrient exports (Ahtiainen and Huttunen 1999; Joensuu et al. 2001; Finér et al. 2010; Futter et al. 2010). However, long-term effects of forestry operations on water courses are still largely unknown. Recent studies indicate considerably longer-term leaching impact from peatland-based forestry to water courses (Nieminen et al. 2017, 2018a). The effects of forest-based bioeconomy management strategies to increase biomass production and its effect on water quality at landscape scale are inadequately understood (Laudon et al. 2011). More knowledge on the impacts of a forest-based bioeconomy on waters is therefore strongly needed, including longer-term datasets and empirical evidence of the catchment and regional-scale impacts from recent shifts towards a forest-based bioeconomy.

Any intensification in forest use can generally be expected to result in increased decomposition of soil organic matter, increased runoff after harvesting and release of nutrients and carbon to waters (Laudon et al. 2011). The most pronounced effects of forestry harvest in mineral soils on surface water quality usually occur at final harvest, i.e. leaching of nitrogen and removal of base cations stored in tree biomass. Soil disturbance and erosion caused by heavy harvesting equipment on wetter soils can lead to higher dissolved organic carbon (DOC) losses and might also promote mercury methylation and runoff of methylmercury (Porvari et al. 2003; Eklof et al. 2016). Previous studies suggest an average increase of one-third in large-scale nutrient loading after forest operations in mineral soils compared with situation before operations (Kortelainen et al. 2006; Tattari et al. 2017). However, there is great local variation and the magnitude of loading is strongly related to local catchment properties and intensity of operations. Oni et al. (2015) have suggested that there are widespread and persistent landscape-scale forestry effects on water quality. The challenge in evaluation of long-term land management practices is that these practices are typically not sufficiently widespread at catchment scale to explain long-term trends in receiving larger water bodies (Kortelainen et al. 1997). Thus, monitoring at all scales from headwaters to large water bodies is needed in order to detect the element load contributions of forestry operations.

In the Nordic countries, a significant proportion of forest biomass is harvested from drained peatlands especially in Finland. In peatland forestry significant loads occur during initial drainage, maintenance operations and especially after final harvest (Kaila et al. 2014). Any future biomass harvesting from peatland forestry areas poses a potential risk to water courses and continuous cover forestry has been suggested to mitigate impacts (Nieminen et al. 2018b). Warm winters result in shorter soil frost period and loading from soft soils can be expected to be increasing after harvesting. Selective harvesting of individual trees within forestry stands maintains sufficient evapotranspiration from stands and local water table level in peat, and thereby reduces the need for drainage network maintenance. Overall, the combined effect of changing climate (temperature, precipitation), increasing peat decomposition due to drainage and fluctuating groundwater levels creates a high risk of nutrient leaching from drained peatlands (Marttila et al. 2018). Considerable area of drained peatland (Laiho et al. 2016) and peaty arable land in the northern Bothnian Bay catchment in Finland create a hot-spot area for potential future increases in nitrogen and carbon loading.

## Climate change imposes additional pressure and modifies loading patterns to Nordic waters

Hydrological conditions and processes in the Nordic region are currently undergoing major changes due to amplified atmospheric and arctic oceanic warming (e.g. Jeppesen et al. 2010). The region is becoming warmer and wetter (Øygarden et al. 2014) and these trends are expected to continue in the future (Arheimer et al. 2005; Huttunen et al. 2015). Especially winters are getting warmer, and springs and autumns wetter, but also getting higher frequency of heavy rains and drought periods (Sorteberg et al. 2018). This is intensifying the hydrological system, accelerating biogeochemical processes and leaching in catchments (Mellander et al. 2018). Higher temperatures, increasing precipitation and changes in the timing and variability of heavy rain events, as well of snow cover and soil frost will all significantly affect the timing and magnitude of nutrients and particle loading from biomass production. Further changes in the timing, seasonality, variability and extreme events of precipitation and temperature are also projected. Apart from affecting the magnitude of loading, climate change will increase the periods when soils are biogeochemically active, thus creating a risk of higher leaching. For example, increased frequency and/or intensity of rain in summer and autumn may increase the risk of erosion and leaching of nutrients. Also higher mineralization of nitrogen due to higher temperature may increase leaching. All these changes in the hydrological cycle will pose challenges to conventional water protection measures and efforts in water protection conducted so far.

Climate change in combination with a focus on increased biomass production may lead to changes in agricultural and forestry management (e.g. increased use of fertiliser), and changes in land use, and therefore overall impacts on water quantity and quality (Rosegrant et al. 2013). Increasing temperature and precipitation will have a positive effect on biomass production systems but simultaneously increase the risk of nutrient and soil losses (Deelstra et al. 2011; Øygarden et al. 2014). Extreme weather events can lead to floods and/or droughts in Nordic catchments, ultimately can accelerate leaching of nutrients to water courses and downstream lakes and coastal waters. Water scarcity may become more common in the Nordic region, as seen in the extremely hot dry summer of 2018, resulting in crop failure and declining groundwater levels. Increased future precipitation during winter and spring, when soil is bare, will also lead to higher runoff and associated nutrient transport and soil losses (Arheimer et al. 2005; Deelstra et al. 2011; Øygarden et al. 2014). Increased nitrogen and phosphorus losses from agricultural sites in Nordic and Baltic countries have already been reported due to changed conditions (Deelstra et al. 2011; Pengerud et al. 2015). In agriculture, soil and nutrient losses during the vegetation-free period will be further accelerated by specific agricultural practices such as autumn ploughing due to increased rain intensity.

Boreal headwaters, lakes and coastal seas are often reported to be the recipient of high carbon and nutrient loads from land, but there are only a few published data on the trends in concentrations in coastal waters (Aksnes et al. 2009). Organic carbon concentrations in many Nordic river basins are rising and total organic carbon fluxes from some river basins to coastal waters are increasing (Fleming-Lehtinen et al. 2015; Räike et al. 2016). Elevated carbon concentrations and brownification are now being detected in all scales (de Wit et al. 2016), from small forested lakes (Vuorenmaa et al. 2006) to large-scale river basins (Lepistö et al. 2008; Räike et al. 2016) and large lakes (Forsius et al. 2017). A warming climate, changes in hydrology and decreases in acidic deposition are considered to be the major driving factors behind trends in carbon export, but are also causing an increasing trend in total nitrogen fluxes (Rankinen et al. 2016). For both forested and agricultural areas in northern parts of the Nordic countries, total nitrogen flux consists mostly of organic nitrogen, whereas at southern sites nitrate-nitrogen dominates in both small upstream catchments and large river basins (Kortelainen et al. 1997; Chen and Bechmann 2019). Overall, a considerable proportion of the nitrogen flux from boreal forest and peatland-dominated river basins may reach lakes and the sea in the form of organic nitrogen. In boreal headwater catchments, carbon and nitrogen losses are highly related to each other because of the dominance of organic nitrogen compounds in nitrogen cycling (Kortelainen et al. 2006; Lepistö et al. 2008).

More sustainable land management is needed to counteract this threat to water quality in streams, lakes and coastal waters. Climate change, nitrogen and phosphorus surplus in agricultural production and atmospheric deposition are current drivers contributing to increasing terrestrial fluxes to the coastal waters, but the bioeconomy and intensified land management is likely to become more important as more biomass is needed. Currently, Nordic assessments on bioeconomy (e.g. Lange et al. 2015) do not sufficiently include environmental impacts of the green shift on watercourses and their quality. For example, environmental impact assessment of new large bioproduct factories are focusing only on its waste receiving waters (e.g. Kile et al. 2019), not on its effects on land use with all consequences.

## Importance of integrated datasets for evaluating the consequences of a Nordic bioeconomy

Nordic countries have a long tradition of monitoring water quality for environmental effects of air quality (e.g. Fölster et al. 2014; De Wit et al. 2016), riverine loading of elements (Skarbøvik et al. 2014; Rankinen et al. 2016; Räike et al. 2019) and their impact on lake eutrophication, agricultural practices (Bechmann et al. 2008; Kyllmar et al. 2014) and to a lesser extent forestry practices (Ahtiainen and Huttunen 1999; Tattari et al. 2017). Effects of forestry and agricultural practices on water quality are also studied in experimental contexts (Lundekvam and Skoien 2007), resulting in complementary insights from the monitoring programmes. Systematic monitoring of forestry impacts on water quality in highly productive forested areas is limited while monitoring in catchments with undisturbed forest has a more extensive geographical coverage. Monitoring of streams and lakes in natural landscapes provides a reference to compare with monitoring of comparable types of rivers and lakes in managed landscapes. Such monitoring of reference sites are essential to answer questions arising about the new pressures to Nordic waters from intensified land use and climate change. There is also a need to incorporate new parameters (emerging pollutants, microplastic, etc.) (e.g. Kaste et al. 2018).

It is not straightforward to predict the impact of climate change and the bioeconomy on water quality for the whole Nordic region. In a European perspective, land cover and land use, geology (Fennoscandian shield with similar bedrock types) and climate show strong similarities for Finland, Sweden and Norway, while Denmark in these respects is more similar to Central-European countries further south and east. However, even within Fennoscandia, large contrasts exist between catchments in terms of landscape (vegetation, land cover, soil type, topography), management and climate (Arheimer et al. 2005). Still, most importantly, the countries are similar enough to benefit from cooperation and knowledge exchange. The form and the degree to which climate change and bioeconomic policy affect water quality will depend on catchment characteristics such as topography, soil, microclimate, land use change and sensitivity of ecosystems. Further, it is unknown how agriculture and forest management will change under various bioeconomic pathways (Rakovic et al. 2020) and how that management will adapt to climate change.

### Long-term continuous datasets

Analysis of long-term datasets is one important way to evaluate and compare the consequences of various actions and measures. However, in long-term datasets the episodic impacts of certain catchment-based measures cannot easily be distinguished from changes, e.g. due to climate, which complicates their interpretation. For example, land use may have long-term influences on water quality (Nieminen et al. 2017). Transport of nutrients from diffuse sources is strongly influenced by a complex combination of temporal and spatial factors, such as fluctuating meteorological and hydrological conditions, geomorphological characteristics, crop cycles and management practices in forestry and agriculture (Palviainen et al. 2013; Kyllmar et al. 2014; Tattari et al. 2017). Spatially, a mosaic of large numbers of different land uses is typical for catchments in the Nordic regions, which can mask the effect of local activities at a larger scale (Oni et al. 2015). Temporally, land uses, and their area vary from year to year, and the impacts of a single practice may last from a couple of years up to one or more decades. Additionally, aquatic processing of nutrients changes the catchment imprint on water quality (de Wit et al. 2018). All these contributing factors make estimation of overall loading complex and challenging at the catchment scale (Haygarth et al. 2012; Bouwman et al. 2013).

### Need for monitoring of small catchments

For reliable future assessments, representative datasets for the various conditions across the entire Nordic region are needed. These need to be acquired on fine time scales, so that ongoing changes and extreme events can be comprehensively detected and assessed. Monitoring data on small representative catchments, experimental plots or river basins loaded mainly by diffuse sources can provide a framework for quantifying and evaluating diffuse source loading of sediments and nutrients. Such data could be used for assessing catchment responses to different land use activities, including activities likely to increase within a bioeconomy framework. For agriculture, field experiments have given knowledge on functioning and risk for nutrient leaching in various crop cultivation systems on different soils and under different climates, which in turn has resulted in recommendations, regulations and subsidies (Bechmann et al. 2016). But knowledge on efficiency of many measures to reduce nutrient leaching is still scarce, e.g. treatment or restored wetlands and buffer zones, use of two stage ditches, liming of clay soils. In forestry, field experiments and paired-catchment studies have increased our understanding of dominant processes and given suggestions for measures. Unfortunately, there are currently insufficient empirical data available on the impacts on water quality of forestry operations used at present, such as whole-tree harvesting, removal of stumps and removal of cutting residues. Recommendation is to establish smart design for monitoring of forestry and agricultural practices, experiments and impacts on water quality. In this context, these recommendations mean using the latest monitoring technologies, existing infrastructure and long time series, combining monitoring efforts from different land use sectors and using representative sites including different risk areas.

While all catchment management activities have potentially severe and undesirable consequences at the local scale, these effects are not readily apparent at the larger landscape scale (Oni et al. 2015). Landscape type is the dominant factor in nutrient and carbon export via boreal rivers. For example, the large changes in carbon flux and nutrient concentration sometime observed in headwater catchments following final felling (Schelker et al. 2012) may be impossible to detect in larger river basins as they represent a relatively small proportion of the total land use pressure. Also, aquatic processes become increasingly important at larger spatial scales, affecting the catchment imprint on water quality. The same applies to other land uses and practices. Thus, location of the monitoring network is key to the successful estimation of leaching and loadings from various land use practices. In addition, lakes play an especially important role in nutrient and carbon cycles and retention, with e.g. more than half of all carbon exported from boreal catchments possibly being consumed in within-lake processes rather than entering the sea (Tranvik et al. 2009). This indicates a need to monitor water quality changes at all scales, in order to detect the true consequences of intensified land use activities.

### Development of new modelling and monitoring tools

Understanding large-scale, complex interactions in Nordic freshwaters is challenging due to the many controlling factors across catchments, climatic conditions, geohydrological and land use practices (Laudon et al. 2011; Futter et al. 2016). Process-based conceptual models are often used to help identify the governing factors for carbon and nutrient dynamics in surface waters at varying scales (Futter et al. 2008; Ledesma et al. 2012). For example, the INCA-C model has been applied to headwater catchments in Fennoscandia (de Wit et al. 2016), to a large boreal river basin in Finland (Lepistö et al. 2014) and to large temperate catchments in Sweden (Ledesma et al. 2012). Previous modelling results obtained using the INCA-C model suggest that climate change-driven patterns in runoff, soil moisture and temperature are typically more important than temporal changes in land management in controlling surface water DOC concentrations. In the forestry sector, annual operations are carried out on only a minor percentage of the catchment area and thus it is challenging to separate catchment-scale impacts of land use activities from the impacts of climate-induced interannual variability (Oni et al. 2015). A decision-support tool for mitigating phosphorus loss from agricultural areas has been widely used by water managers to optimise implementation of mitigation measures in Norway (Drohan et al. 2019). This model was validated on long-term monitoring data for small agricultural catchments. However, in the agricultural sector, results obtained using the INCA-P model have shown that land use change is more effective than changes in agricultural practices in controlling phosphorus losses (Farkas et al. 2013). Another widely used model is the Soil & Water Assessment Tool (SWAT or SWAT+), which has been successfully applied to various land use practices in the Nordic region (e.g. Hashemi et al. 2016). Many of the Nordic countries also have their own national models that can be used to model the effects of large-scale changes in land use patterns. The results are used for reporting to EU, HELCOM and for national environmental goal assessments, but not yet to model scenarios of an increased future reliance on the bioeconomy from the water quality point of view.

Overall, future monitoring efforts should seek to include new monitoring methods and modelling tools. This would provide more information about the governing factors and processes, and also allow more accurate prediction of future scenarios. For example, sensors offer continuous monitoring of water quality that can give increased understanding of pollutant transport during extreme events, something that is more difficult to detect from infrequent grab sampling or composite sampling (e.g. Koskiaho et al. 2010; Skarbøvik and Roseth 2014). Results from different parts of the Nordic region obtained using multiple modelling tools could be combined. Availability of long-term continuous datasets and data on representative small catchments across the Nordic region would then help to focus more intensively on current and future scenarios in different perspectives. Monitoring systems for land management and water quality and quantity, in combination with hydrological and ecological modelling, could give an indication about the future situation and provide knowledge on feasible mitigation measures and adaption strategies (Giri and Qiu 2016; Couture et al. 2018).

### Can integrated, knowledge-based land use planning help mitigate leaching?

Land use planning is a complicated process where multiple and often controversial objectives have to be taken into account. Awareness of the immaterial benefits humans derived from nature is increasing, so new approaches are required to incorporate impacts on various ecosystem services into land use planning (Tolvanen et al. 2013; Johansen et al. 2018). A well-known example is the conflict between efficient agricultural production and environmental goals. Furthermore, for example recent studies on drained low-productivity peatlands in Finland found strong trade-offs between biodiversity, nutrient loading to waters and greenhouse gas balances. These trade-offs are also strongly variable over time (Tolvanen et al. 2018; Juutinen et al. 2019, 2020) and imply that different targets cannot be achieved simultaneously. This means that the choice of optimal land use options requires compromises, case-specific assessments and consideration of the duration of effects in order to balance multiple conflicting objectives (Kurttila et al. 2020). For accurate evaluation of these trade-offs and consequences, data obtained through proper long-term monitoring and modelling of surface waters in a catchment perspective are essential.

Combining know-how on reducing nutrient and carbon leaching, monitoring data and modelling results in multiple land use planning would offer a powerful tool to optimise land use effects from several points of views. Trade-offs between ecosystem services and effects on water bodies cannot always be completely avoided, but they can be reduced with careful land use planning. For example, previous land use and land management optimisation studies have found that relatively high environmental benefits can be achieved with a low reduction in economic returns (Pennington et al. 2017). However, while the initial increases in environmental benefits can be inexpensive, further efforts may increase the costs considerably (Juutinen et al. 2019). Numerical multi-objective optimisation can be a useful tool in identifying cost-effective land use and land management approaches that simultaneously supply ecosystem services and economic returns at landscape or regional level (Johansen et al. 2018) with further links to water quality from different land uses. In the future we need to develop decision-support systems, where water quality monitoring and modelling are an integrated part.

## Future pathways and recommendations for a sustainable bioeconomy in terms of water quality

From a bioeconomy perspective, the pressures on water quality are related to changes in the intensity of agriculture and forestry. To what extent this affects water quality is difficult to assess, whether the pressures primarily act on a local scale, in headwaters, or on a regional scale in downstream rivers and lakes, or all the way to marine coastal ecosystems. This is partly because land use impacts generating diffuse loading interact with other anthropogenic pressures such as point-source pollution, atmospheric deposition of nitrate, hydromorphological alteration habitats and climate change. Also, aquatic processes become increasingly important at larger spatial scales, impacting the catchment imprint on water quality. In concert, multiple anthropogenic pressures—modified by aquatic processing—(potentially) threaten the ecological status of surface waters (EEA 2018). Disentangling and quantifying the cause-and-effect relationship between multiple pressures and ecological functioning is challenging, especially when addressing the regional scale (Birk et al. 2020). To understand effects of land use on water quality, we need (i) monitoring, (ii) modelling, (iii) experiments and (iv) expert judgement. Especially combining monitoring data and modelling is required to support integrated, knowledge-based land use planning.

Monitoring data from small, well-defined, data-rich and well-understood catchments have great potential to provide insights into cause–effect relationships between multiple pressures and water quality. The results of such analysis would give a good understanding of the effects of various measures and thus assist in regional- and Nordic-scale land use planning. This would in turn help tackle the future challenge of water quality under threat from the green shift in Nordic catchments. To mitigate effects of intensified land use, it is critical to have efficient monitoring programmes that enable detection of changes and selection of the most efficient countermeasures. To be able to distinguish water quality responses from local activities within the catchment and responses from external widespread stressors (e.g. deposition and climate change), an understanding of reference water quality is needed. For understanding and quantifying reference water quality, continuation of long-term monitoring of natural catchments is necessary. However, simultaneously we need smart and systematic monitoring of land use impacts. Combination of long-term reference sites with land-use-impacted catchments having comparable types of rivers and lakes (the paired-catchment approach) is recommended focusing on modern land use changes across the Nordic region. Different nature of impacts in agriculture and forestry are needed to take account as time horizon from loading perspective differs in agricultural and forestry operations and thus require a different design.

The Nordic countries have a long tradition in knowledge-based management of natural resources. However, increasing land use pressures call for new sustainable solutions for land use management. For example, to date conventional measures to control nutrient loading have focused on (i) management methods in fields or forest or (ii) ‘end-of-pipe’ solutions where outgoing waters are directed through various water protection measures such as settling basins. Especially end-of-pipe methods have been shown to be inefficient in many cases and it has been pointed out that the focus should be on prevention of processes leading to leaching and erosion, rather than treating water post contamination (Nieminen et al. 2018c).

The main knowledge gap in the Nordic countries related to effect of intensified land management on water quality is lack of a holistic understanding of driving processes at different scales from headwaters to lakes and to coastal waters, including the role of aquatic processing (e.g. N-retention in wetlands, P-retention in lakes, food-web interactions) for modifying the pressures and impacts on water quality. We propose the following actions for better assessment of bioeconomy-associated impacts and improvement of mitigation efforts to reduce nutrient loading to Nordic waters.

- Sustain or improve current monitoring programmes by developing a cost-efficient monitoring allowing a more systematic assessment of the impacts of forestry and agriculture on water quality, using unmanaged sites as reference (“paired-catchment approach”).
- Development of monitoring and modelling tools including databases to assess temporal change in responses to single-event interventions (such as forest harvest, forest fertilisation or establishment of buffer zones).
- Extend existing monitoring programmes to include new parameters needed for modern management choices.
- Promote integration of monitoring programmes operating at different catchment scales and of different water body types (streams, rivers, lakes), with special attention for the use and development of catchment models and understanding of aquatic processing.
- Improved management and availability of national and international datasets with open-access data sharing.
